# A novel interpretative tool for early prediction of low cardiac output syndrome after valve surgery: online machine learning models

**DOI:** 10.1080/07853890.2023.2293244

**Published:** 2023-12-21

**Authors:** Liang Hong, Tianling Feng, Runze Qiu, Shiteng Lin, Yinying Xue, Kaizong Huang, Chen Chen, Jiawen Wang, Rongrong Xie, Sanbing Song, Cui Zhang, Jianjun Zou

**Affiliations:** a Cardiovascular Intensive Care Unit, Department of Critical Care Medicine, Nanjing First Hospital, Nanjing Medical University, Nanjing, China; b School of Basic Medicine and Clinical Pharmacy, China Pharmaceutical University, Nanjing, China; c Department of Clinical Pharmacology, Nanjing First Hospital, Nanjing Medical University, Nanjing, China; d Department of Pharmacy, Nanjing First Hospital, China Pharmaceutical University, Nanjing, China; e School of Pharmacy, Nanjing University of Chinese Medicine, Nanjing, China

**Keywords:** Low cardiac output syndrome, valve surgery, machine learning, prediction, interpretative, online

## Abstract

**Objective:**

Low cardiac output syndrome (LCOS) is a severe complication after valve surgery, with no uniform standard for early identification. We developed interpretative machine learning (ML) models for predicting LCOS risk preoperatively and 0.5 h postoperatively for intervention in advance.

**Methods:**

A total of 2218 patients undergoing valve surgery from June 2019 to Dec 2021 were finally enrolled to construct preoperative and postoperative models. Logistic regression, support vector machine (SVM), random forest classifier, extreme gradient boosting, and deep neural network were executed for model construction, and the performance of models was evaluated by area under the curve (AUC) of the receiver operating characteristic and calibration curves. Our models were interpreted through SHapley Additive exPlanations, and presented as an online tool to improve clinical operability.

**Results:**

The SVM algorithm was chosen for modeling due to better AUC and calibration capability. The AUCs of the preoperative and postoperative models were 0.786 (95% CI 0.729–0.843) and 0.863 (95% CI 0.824–0.902), and the Brier scores were 0.123 and 0.107. Our models have higher timeliness and interpretability, and wider coverage than the vasoactive-inotropic score, and the AUC of the postoperative model was significantly higher. Our preoperative and postoperative models are available online at http://njfh-yxb.com.cn:2022/lcos.

**Conclusions:**

The first interpretable ML tool with two prediction periods for online early prediction of LCOS risk after valve surgery was successfully built in this study, in which the SVM model has the best performance, reserving enough time for early precise intervention in critical care.

## Introduction

Low cardiac output syndrome (LCOS), defined as requiring at least two inotropes or mechanical circulatory support 24–48 h after surgery [1], is one of the most common and risky postoperative complications of valve disease in the intensive care unit (ICU) due to the dramatic increase in afterload [2–4]. During LCOS, the sympathetic hyperexcitability from myocardial injury and diminished cardiac output causes insufficient peripheral perfusion, microcirculation disorders, and ultimately multiorgan failure [5]. Administration of two or more inotropes is the preferred regimen for LCOS [6], but there has been no reliable data to support any inotropes that reduce mortality in patients with LCOS [7,8]. Patients with LCOS not controlled by inotropes require an intra-aortic counterpulsation balloon pump, left ventricular assist device, or extracorporeal membrane oxygenation for mechanical circulatory support within five days after surgery [6], portending poor outcomes. LCOS is reversible at an early stage, so early identification is necessary to improve prognosis.

Traditional diagnostic methods have limitations in the early or precise prediction of LCOS. Transthoracic and transesophageal echocardiography are the tools of choice for evaluating LCOS [9], but they cannot continuously and accurately measure hemodynamics, and are extremely operator-dependent [10]. The pulmonary artery catheters (PAC) and pulse contour analysis, such as PiCCO, LiDCO and FloTrac [11], can continuously and sensitively monitor cardiac output, but their invasive and costly properties limit the application in routine preoperative diagnosis, and instead, they are performed intraoperatively. Non-invasive real-time hemodynamic monitoring only provides auxiliary diagnosis for invasive devices [12]. The laboratory examination is also acknowledged as a means of early assessment. Conventional indicators such as lactate and bilirubin are reported as independent risk factors for LCOS [13,14], but which indicators have predictive value remains inconclusive. Predictive models can overcome the shortcomings of such diagnostic modalities, enabling early and accurate identification. Machine learning (ML) models, which have been applied in LCOS prediction in our past research [15], make predictions by learning from enormous data without specific programming, and the results are more considerate and accurate than programming models, especially for large amounts of data in ICU [16,17]. The present study, on the other hand, predicts LCOS risk after valve surgery, based on richer data.

**Figure 1.**
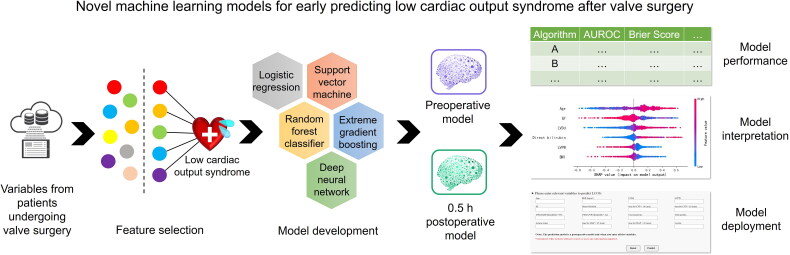
Graphical abstract. We developed novel machine learning models for early predicting low cardiac output syndrome after valve surgery preoperatively and 0.5 h postoperatively.

This retrospective study aims to develop the first interpretative ML model to early predict LCOS risk after valve surgery, with clinically friendly variable input modules and understandable risk assessment results. The model incorporated preoperative, intraoperative and postoperative parameters, and contained two prediction periods, preoperatively and 0.5 h postoperatively (Figure 1). The clinical application of this tool will timely predict the LCOS risk during postoperative critical care, allowing sufficient time for clinical intervention, which improves patient outcomes and reduces the occupation of medical resources.

## Methods

### Study population

We collected the data from a total of 2304 patients who underwent valve surgery in the ICU of Nanjing First Hospital from June 2019 to Dec 2021 from electronic medical records and physiological monitors. Patients who received valve surgery during the study period were recruited as the study objects, including but not limited to aortic and/or mitral heart valve surgery, both repair and replacement. Patients were excluded based on the following criteria: (1) Age <18; (2) Death during or within 6 h after surgery; (3) Lack of important variables. A total of 2218 patients were retrospectively included. The ethics committee of the Nanjing First Hospital has approved this study (Grant No. KY20220518-KS-01) on 18 May 2022 and waived the need for informed consent.

### Characteristics

Variables at different stages from patients undergoing valve surgery with postoperative LCOS were collected, including preoperative variables such as sex, age, BMI, laboratory indicators, echocardiographic parameters (e.g. EF, LVDd, LVPW) and intraoperative variables including cardiopulmonary bypass (CPB) time, aortic clamping time (actime), and calculated hemodynamic variables. Hemodynamic variables including mean arterial pressure (MAP), systolic blood pressure (SBP), and diastolic blood pressure (DBP) were integrated by the machine into the existing system every 5 min intraoperatively and saved as time-series data. In order to comprehensively evaluate the time-series data, we calculated the following variables from the data automatically recorded by the intraoperative monitor based on a previous study [18]. (1) Time for MAP <65, 60, 55, and 50; (2) Time for central venous pressure (CVP) >12, 16, and 20; (3) Area under the curve for MAP below the threshold (AUT, the thresholds were set at 65, 60, 55, and 50); (4) Area under the Curve (AUC) for central venous pressure (CVP) below the threshold (AUT-CVP, the thresholds were set at 65, 60, 55, and 50); (5) Time-weighted AUT (TWA-MAP, the thresholds were set at 65, 60, 55, and 50); (6) Time-weighted AUT-CVP (TWA-CVP, the thresholds were set at 12, 16, and 20). Lactate was collected 0.5 h postoperatively.

### Outcomes

The primary outcome was the diagnosis of LCOS when one or more of the following criteria were satisfied. (1) Cardiac index reduced to <2.2 L/min/m^2^; (2) Systolic blood pressure <90 mmHg with signs of tissue hypoperfusion, including oliguria (urine output <1 ml/kg·h), and/or elevated lactate level >3.0 mmol/L; (3) Requiring mechanical circulatory support or inotropic agents (dopamine or dobutamine at least 4 µg/kg·min for ≥12 h, and/or epinephrine at least 0.02 µg/kg·min, and/or milrinone at least 0.2 µg/kg·min, and/or levosimendan at least 0.05 µg/kg·min) to maintain hemodynamics after optimizing preload. Patients receiving vasoconstricting medications to increase peripheral vascular resistance at normal cardiac output were not considered to have LCOS.

### Statistical analysis

Statistical analysis was performed using SPSS version 25.0 (IBM Corporation, Armonk, NY, USA) to compare the differences in baseline characteristics between patients with and without a diagnosis of LCOS. The normal distribution of all continuous variables was evaluated using the Shapiro-Wilk test. Continuous variables were expressed as mean (standard deviation) or median (interquartile range), while categorical variables were expressed as percentages. Continuous variables were analyzed using the Student’s *t*-test or Mann-Whitney *U*-test, and categorical variables were analyzed using the chi-square test or Fisher exact test. *p* < .05 (two-sided) was considered statistically significant.

### Data pre-processing

Variables containing more than 10% of the missing data were removed, and missing values were subsequently supplemented according to the k-nearest neighbor algorithm [19]. All continuous variables were normalized using *Z*-score normalization [20], and One-Hot encoding converted multiple categorical variables [21]. The dataset was randomly divided into training and testing cohorts in a ratio of 8:2, with a similar proportion of patients with LCOS in each cohort. The training cohort was utilized for feature selection, model training, and parameter tuning, while the test cohort was used only as an internal validation cohort to evaluate the generalization ability of the model internally.

### Feature selection

Redundancies and irrelevant factors in the dataset may lead to overfitting, and reduce the predictive performance of ML models, so feature selection is necessary. Preoperative and baseline variables were included in the Least Absolute Shrinkage and Selection Operator (LASSO) regression for early assessment of LCOS risk in patients undergoing valve surgery. By adjusting the hyperparameter lambda (λ), the LASSO algorithm shrinks all regression coefficients to zero, and excludes extraneous features by making their coefficients exact to zero. The larger the absolute value of the regression coefficients, the more significant the relationship between the features and outcomes. Thus, variables with nonzero coefficients determined by the LASSO algorithm were incorporated for constructing ML models. The feature selection algorithm was implemented with the Python package Scikit-learn (version 0.23.2).

### Model development

Five ML classifiers including logistic regression (LR), support vector machine (SVM), random forest classifier (RFC), extreme gradient boosting (XGB), and deep neural network (DNN) were executed for model construction to predict LCOS in patients undergoing valve surgery. In the training cohort, we constructed two models to predict preoperatively and 0.5 h postoperatively. A cost-sensitive learning approach was used to reduce patient misclassification accounting for the potential impact of unbalanced data on model training. Hyperparameter optimization for each model was optimized by a grid search algorithm and 10-fold cross-validation for the highest AUC of the receiver operating characteristic (ROC).

### Model evaluation

The predictive performance of the model was mainly evaluated by the AUC score of ROC on the test cohort. The ROC curves of different models were compared by DeLong’s test, and the sensitivity, specificity, and accuracy of each model were calculated at the optimal threshold determined by the maximum Youden index (sensitivity + specificity − 1) on the ROC curves. The calibration performance of the models was evaluated based on the calibration curves and Brier scores. The optimal model was selected by considering both predictive and calibration capabilities. In addition, we compared the predictive performance of the preoperative and postoperative models with the vasoactive-inotropic score (VIS) [22], a tool for clinical assessment of LCOS.

### Model interpretation

Uninterpretability is a disadvantage of traditional ML models, which means that the training and validation of ML models are equivalent to being packed in a black box. Therefore, we introduced the SHapley Additive exPlanations (SHAP) method to improve the interpretability and visualization of the models. SHAP values quantify the association between a variable and the marginal contribution to an individual’s final risk prediction, and the average absolute SHAP value for all patients is reported as the SHAP value of a variable.

### Model deployment

The pickle package in python was used to save and load our preoperative and postoperative models. The back-end server-side was written in Flask, a framework for serving Python web applications. The web application of our model was deployed on the Nginx server.

## Results

### Population characteristics

**Figure 2.**
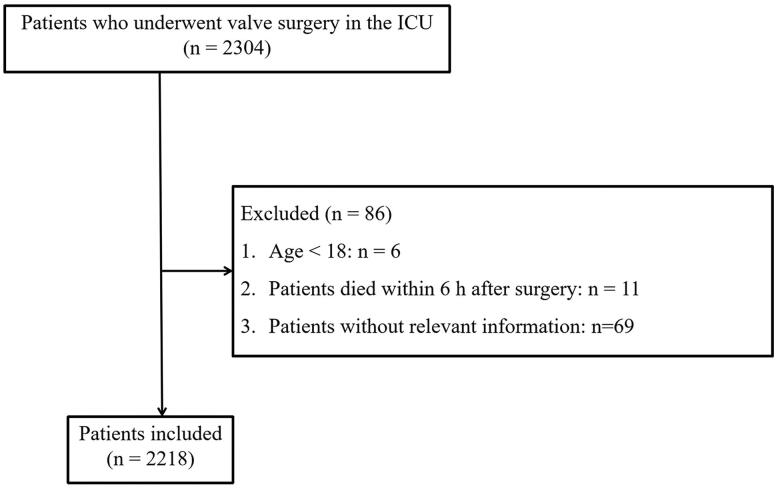
The flow chart for enrollment and exclusion of patients with low cardiac output syndrome.

Among 2218 patients included in this study, 400 patients suffered from LCOS. An inclusion and exclusion flow chart is shown in Figure 2. Six patients were younger than 18 years old at the onset, 11 patients died within 6 h after surgery, and 69 patients without relevant information were excluded. The median age of enrolled patients was 63 (interquartile range, 54–70), and 56.4% were male. The baseline statistics for the groups with and without LCOS are depicted in Table 1. Patients with LCOS and those without LCOS were not significantly different in Emergency admission and Time-weighted AUT (TWA-MAP, the thresholds were set at 65, 60, 55, and 50) (*p* > .05).

**Table 1.** Demographics and clinical characteristics of the patients.

Variables	Total (*n* = 2218)	Non-LCOS (*n* = 1818)	LCOS (*n* = 400)	*p*-value
Baseline characteristics				
Age, years	63 (54–70)	62 (53–69)	66 (58–72)	<.001
Male, %	1253 (56.5)	991 (54.5)	262 (65.5)	<.001
BMI, kg/m^2^	23.63 (21.45–25.95)	23.73 (21.63–25.95)	22.86 (20.70–25.71)	<.001
Emergency admission, %	106 (4.8)	82 (4.5)	24 (6.0)	.206
Operation time, h	4.08 (3.42–5.00)	4.00 (3.33–4.83)	4.58 (3.92–5.58)	<.001
Cpbtime, min	113 (80–147)	110 (78–142)	133 (95–166)	<.001
Actime, min	82 (60–108)	79 (58–104)	97 (72–122)	<.001
Laboratory indicators				
Lactate, mmol/L	1.8 (1.1–2.9)	1.7 (1.1–2.5)	3.1 (1.8–4.9)	<.001
Alanine aminotransferase, µ/L	18 (13–28)	18 (13–27)	20 (14–31)	.017
Creatinine, μmol/L	72.50 (60.98–87.00)	71.15 (60.00–85.00)	78.30 (66.00–95.98)	<.001
Indirect bilirubin, μmol/L	9.0 (6.3–12.0)	8.8 (6.2–11.7)	9.6 (7.0–13.9)	<.001
Lymphocytes,10^9^/L	26.80 (20.88–33.00)	27.20 (21.30–33.50)	24.60 (18.90–30.30)	<.001
Urea, mmol/L,	6.45 (5.20–8.00)	6.30 (5.10–7.90)	7.13 (5.79–8.90)	<.001
Globulin, g/L	26.40 (23.90–29.50)	26.50 (24.10–29.50)	25.92 (23.50–29.18)	.022
Platelet count,10^9^/L	170 (135–213)	171 (137–215)	160 (128–201)	.003
Direct bilirubin, μmol/L	3.1 (2.3–4.6)	3.0 (2.2–4.4)	3.9 (2.8–6.3)	<.001
Neutrophils, 10^9^/L	61.8 (55.1–68.2)	61.3 (54.7–67.9)	63.5 (57.8–70.2)	<.001
Total bilirubin, μmol/L	12.3 (8.9–16.9)	12.0 (8.6–16.3)	13.9 (10.4–20.3)	<.001
Total protein, g/L	66.6 (62.7–70.8)	66.9 (63.2–71.0)	65.1 (60.9–69.0)	<.001
Hemodynamic variables				
time for MAP <65, min	125 (90–175)	120 (90–165)	160 (115–220)	<.001
time for MAP <60, min	90 (60–130)	90 (60–125)	120 (76–169)	<.001
time for MAP <55, min	65 (40–95)	60 (35–90)	80 (45–120)	<.001
time for MAP <50, min	35 (20–60)	35 (20–55)	45 (25–75)	<.001
time for CVP >12, min	20 (5–60)	15 (5–55)	38 (5–85)	<.001
time for CVP >16, min	0 (0–10)	0 (0–10)	5 (0–30)	<.001
time for CVP >20, min	0 (0–5)	0 (0–0)	0 (0–10)	<.001
AUT (thresholds =65)	1500 (975–2165)	1440 (925–2046)	1830 (1204–2638)	<.001
AUT (thresholds =60)	910 (555–1385)	880 (525–1325)	1123 (678–1750)	<.001
AUT (thresholds =55)	500 (275–820)	488 (260–795)	588 (330–1010)	<.001
AUT (thresholds =50)	240 (110–430)	230 (105–425)	275 (135–516)	<.001
AUT-CVP (thresholds =12)	60 (5–230)	50 (5–200)	140 (20–458)	<.001
AUT-CVP (thresholds =16)	0 (0–60)	0 (0–50)	15 (0–194)	<.001
AUT-CVP (thresholds =20)	0 (0–10)	0 (0–0)	0 (0–53)	<.001
TWA-MAP (thresholds =65))	11.67 (9.54–13.94)	11.70 (9.50–13.96)	11.54 (9.77–13.73)	.936
TWA-MAP (thresholds =60)	9.78 (7.95–11.93)	9.87 (8.00–11.94)	9.51 (7.81–11.92)	.352
TWA-MAP (thresholds =55)	8.11 (6.46–10.00)	8.16 (6.49–10.00)	7.85 (6.39–9.65)	.211
TWA-MAP (thresholds =50)	6.53 (4.90–8.27)	6.57 (4.82–8.30)	6.40 (5.00–8.08)	.740
TWA-CVP (thresholds =12)	2.22 (1.00–4.32)	2.00 (1.00–4.00)	2.90 (1.50–6.00)	<.001
TWA-CVP (thresholds =16)	0.00 (0.00–3.23)	0.00 (0.00–3.00)	1.55 (0.00–5.20)	<.001
TWA-CVP (thresholds =20)	0.00 (0.00–1.00)	0.00 (0.00–0.00)	0.00 (0.00–3.83)	<.001
Echocardiographic parameters				
EF, %	62 (57–64)	62 (59–64)	55 (43–61)	<0.001
LVDd, mm	56 (49–62)	55 (48–61)	62 (54–68)	<0.001
LVPW, mm	9.55 (9.00–10.30)	9.80 (9.00–10.50)	9.00 (9.00–10.00)	<0.001

Male and emergency admission are represented as number (%), and other variables are represented as median (interquartile range). LCOS: Low cardiac output syndrome; BMI: body mass index; Cpbtime: cardiopulmonary bypass time; Actime: Aortic clamping time; MAP: mean arterial pressure; CVP: central venous pressure; AUT: area under the curve for blood pressure below the threshold; AUT-CVP: area under the curve for central venous pressure above the threshold; TWA-MAP: time weighted area under the curve for mean arterial pressure below the threshold; TWA-CVP: time weighted area under the curve for central venous pressure above the threshold; EF: ejection fraction; LVDd: left ventricular diastolic diameter; LVPW: left ventricular posterior wall thickness.

### Feature selection

**Figure 6.**
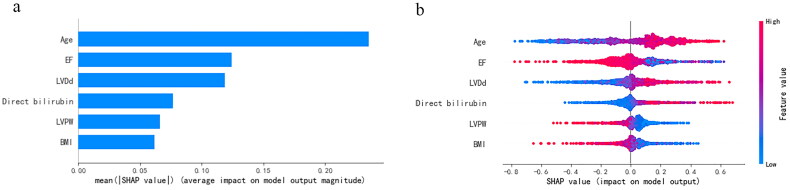
The ranking of feature weights based on Shapley Additive exPlanations (SHAP) values in the preoperative model. (a) The standard bar chart shows the SHAP values for each feature and was sorted by weight. (b) In the scatter plot of feature density, red dots indicate higher value, and blue dots indicate lower value. More red dots on the right side of the vertical axis indicate positive correlation with low cardiac output syndrome, while more red dots on the left side indicate negative correlation. EF: ejection fraction; LVDd: left ventricular diastolic diameter; LVPW: left ventricular posterior wall thickness; BMI: body mass index.

**Figure 7.**
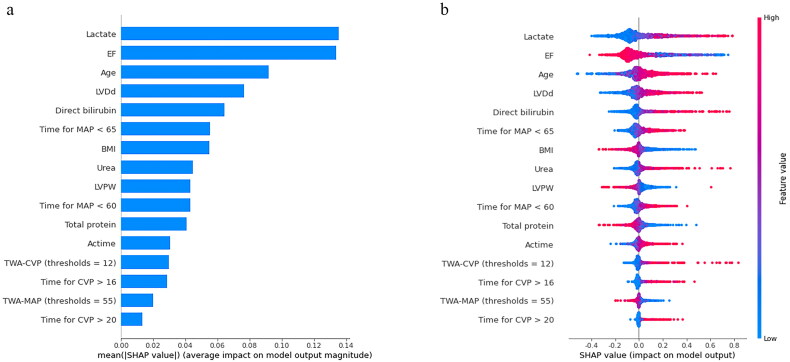
The ranking of feature weights based on Shapley Additive exPlanations (SHAP) values in the postoperative model. (a) The standard bar chart shows the SHAP values for each feature and was sorted by weight. (b) In the scatter plot of feature density, red dots indicate higher value, and blue dots indicate lower value. More red dots on the right side of the vertical axis indicate positive correlation with low cardiac output syndrome, while more red dots on the left side indicate negative correlation. EF: ejection fraction; LVDd: left ventricular diastolic diameter; MAP: mean arterial pressure; BMI: body mass index; LVPW: left ventricular posterior wall thickness; actime: aortic clamping time; CVP: central venous pressure; TWA-CVP: time weighted area under the curve for central venous pressure above the threshold; TWA-MAP: time weighted area under the curve for mean arterial pressure below the threshold.

All preoperative variables were included in the LASSO regression for feature selection. The preoperative model outputted six variables, including age, ejection fraction (EF), left ventricular diastolic diameter (LVDd), direct bilirubin, left ventricular posterior wall thickness (LVPW), and BMI (Figure 6(a)). In addition, all preoperative, intraoperative, and postoperative baseline variables were included in the LASSO regression to construct the postoperative model. Finally, variables including lactate, EF, age, LVDd, direct bilirubin, time for MAP <65, BMI, urea, LVPW, time for MAP <60, total protein, actime, TWA-CVP (thresholds = 12), time for CVP >16, TWA-MAP (thresholds = 55), and time for CVP >20 were finally incorporated into the postoperative model (Figure 7(a)).

### Model performance

**Figure 3.**
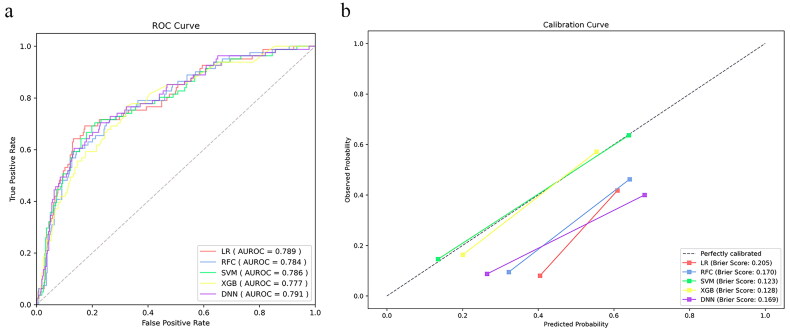
The prediction and calibration capability of the preoperative machine learning model for predicting low cardiac output syndrome. (a) The receiver operating characteristic curve (ROC) for the testing cohort. (b) The calibration curve for the area under the curve of the testing cohort. AUROC: area under the ROC; LR: logistic regression; SVM: support vector machine; RFC: random forest classifier; XGB: extreme gradient boost; DNN: deep neural network.

**Figure 4.**
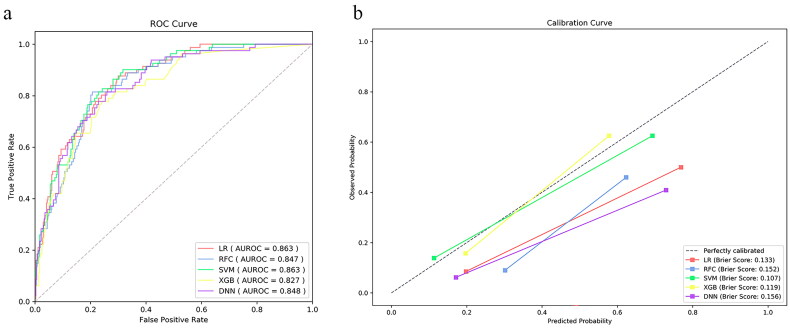
The prediction and calibration capability of the postoperative machine learning model for predicting low cardiac output syndrome. (a) The receiver operating characteristic curve (ROC) for the testing cohort. (b) The calibration curve for the area under the curve of the testing cohort. AUROC: area under the ROC; LR: logistic regression; SVM: support vector machine; RFC: random forest classifier; XGB: extreme gradient boost; DNN: deep neural network.

**Figure 5.**
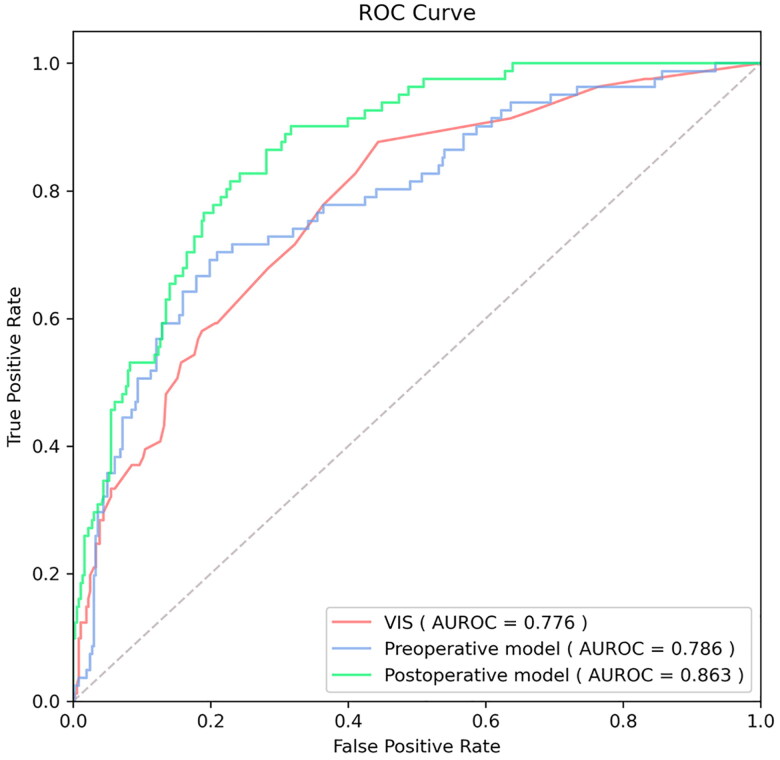
The receiver operating characteristic curve (ROC) of vasoactive-inotropic score (VIS), and preoperative and postoperative machine learning models. AUROC: area under the ROC.

We applied five different algorithms, including LR, RFC, SVM, XGB and DNN, to construct ML models and evaluated their performance to predict LCOS occurrence using AUCs and Brier scores. The AUC and Brier scores of the preoperative model using the SVM algorithm were 0.786 (95% CI 0.729–0.843) and 0.123 (Figure 3(a,b)), exhibiting the highest AUC and best calibration capability among the five algorithms. Although the SVM algorithm did not show the highest discriminative performance, this parameter did not differ much between the five algorithms. Consequently, SVM was adopted as the algorithm for the final prediction model. The AUC and Brier scores of the postoperative model based on SVM were 0.863 (95% CI 0.824–0.902) and 0.107 (Figure 4(a,b)), exhibiting good performance. In addition, we compared the SVM model to the VIS score that is most commonly used to evaluate LCOS at present. The AUC of the VIS score was 0.776 (95% CI 0.721–0.831), which was significantly lower than the postoperative model (Table 2 and Figure 5).

**Table 2.** Comparison of performance of VIS with preoperative and postoperative models.

LCOS Assessment Tool	AUC (95% CI)	*p*-value
VIS	0.776 (0.721–0.831)	
Preoperative model	0.786 (0.729–0.843)	.7997
Postoperative model	0.863 (0.824–0.902)	.0033

VIS: vasoactive-inotropic score; LCOS: low cardiac output syndrome; AUC: area under the curve of receiver operating characteristic; CI: confidence interval.

The AUC, sensitivity, specificity, accuracy, and Brier score of the testing cohort for preoperative and postoperative models were provided in Tables 3 and 4, respectively, and Supplementary Figures 1 and 2 indicated the discriminative performance on the training cohort of the preoperative and postoperative models, respectively. The SVM model achieved the best specificity (76.6%), accuracy (75.7%) and Brier score (0.123) in predicting LCOS among the preoperative ML models, and AUC is second only to LR (0.786 vs 0.789). The SVM model showed the best specificity (83.5%), accuracy (80.6%), Brier score (0.107) and AUC (0.863) for predicting LCOS among the postoperative ML models. The discriminative performance was observed in the testing cohort of the preoperative model (Figure 3(a)). There was no statistical difference in the AUCs by DeLong’s test. Figure 3(b) presented calibration curves and the Brier score of preoperative ML models in the testing cohort. Similarly, Figure 4(a) exhibits the ROC of the postoperative models, and Figure 4(b) shows the calibration curve of the postoperative models.

**Table 3.** Prediction and calibration performance of the five algorithms in the testing cohort of preoperative model.

Algorithm	AUC (95% CI)	Sensitivity	Specificity	Accuracy	Brier score
LR	0.789 (0.733–0.846)	71.6%	74.4%	73.9%	0.205
RFC	0.784 (0.729–0.839)	75.3%	66.4%	68.0%	0.170
SVM	0.786 (0.729–0.843)	71.6%	76.6%	75.7%	0.123
XGB	0.777 (0.721–0.832)	72.8%	68.6%	69.4%	0.128
DNN	0.791 (0.735–0.847)	70.4%	74.4%	73.6%	0.169

AUC: area under curve of receiver operating characteristic; CI: confidence interval; LR: logistic regression; RFC: random forest classifier; SVM: support vector machine; XGB: extreme gradient boosting; DNN: deep neural network.

**Table 4.** Prediction and calibration performance of the five algorithms in the testing cohort of postoperative model.

Algorithm	AUC (95% CI)	Sensitivity	Specificity	Accuracy	Brier score
LR	0.863 (0.823–0.902)	71.6%	81.8%	80.0%	0.133
RFC	0.847 (0.806–0.889)	81.5%	75.8%	76.8%	0.152
SVM	0.863 (0.735–0.856)	67.9%	83.5%	80.6%	0.107
XGB	0.827 (0.779–0.874)	65.4%	80.2%	77.5%	0.119
DNN	0.848 (0.805–0.892)	76.5%	77.4%	77.3%	0.156

AUC: area under curve of receiver operating characteristic; CI: confidence interval; LR: logistic regression; RFC: random forest classifier; SVM: support vector machine; XGB: extreme gradient boosting; DNN: deep neural network.

### Model Interpretation

We introduce the SHAP algorithm to visually interpret the predictions of the best-performing SVM model in the previous evaluation. Figures 6(a) and 7(a) are feature importance ranking graphs drawn according to the average absolute values of feature SHAP values in the model. According to the SHAP value, the three most important features in the preoperative model were age, EF, and LVDd (Figure 6(a)), and the eight features in the postoperative model were EF, lactate, age, LVDd, direct bilirubin, BP_65_time, BMI, and urea in order of impact (Figure 7(a)). Figures 6(b) and 7(b) are beeswarm plots of SHAP value distributions of various features included in the model. The redder the color of the sample point led to a higher value of the sample variable and high SHAP values resulted in a higher risk of LCOS. During preoperative prediction, the older the age, the lower the EF, and the larger the LVDd, the more likely the patient was to suffer from LCOS (Figure 6(b)). In the postoperative model, patients with lower EF, higher lactate, older age, larger LVDd, higher direct bilirubin, and longer time of MAP <65 were at higher risk for LCOS (Figure 7(b)).

### Online application

Finally, we deployed our preoperative and postoperative ML models on web pages for easy use by clinicians and researchers, which are available at http://njfh-yxb.com.cn:2022/lcos. The probability of a patient suffering LCOS after valve surgery was successfully displayed by inputting the relevant variables of LCOS.

## Discussion

As a complication of valve surgery with an morbidity of up to 45% [23] and 30-day mortality of 38% [2], LCOS lacks a predictive model based on ML. In this retrospective study, the predictive tool we developed is the first application of ML technology for LCOS prediction. The tool incorporated multi-period variables such as echocardiography, laboratory examination, and hemodynamic monitoring, which are readily accessible general parameters. According to the acquisition time of the variables, we divided the tool into preoperative and 0.5 h postoperative models. Furthermore, the models were interpreted via SHAP, and visualized online for physicians to operate and make decisions at the bedside. Our predictive tool could increase the early intervenability of LCOS.

### The preoperative model allows early intervention

We integrated multiple preoperative features in the preoperative model. Among the output variables, age and BMI were obtained at admission, while other parameters were derived from laboratory examination and echocardiography 48–72 h before operation. Advanced age and low LVEF (<40%) were reported independent predictors of LCOS after valve surgery [24,25], which accounted for the largest proportion in our preoperative model. LVEF is a widely used tool to assess left ventricular systolic function, with lower LVEF indicating an increased risk of LCOS [26,27]. Increased LVDd, another risk variable in the model, implies myocardial dilation, which was an independent predictor of sudden cardiac death, and the incidence further increased when combined with a decline in EF [28]. Postoperative bilirubin was reported as an independent predictor [14], but direct bilirubin measured preoperatively is already an important risk factor for predicting LCOS in our model. Low BMI (<20 kg/m^2^) was also reported to be associated with LCOS [29], which is consistent with our results.

Due to the early access to these variables, our preoperative model provided an ultra-early prediction of LCOS, properly classifying patients by risk preoperatively. For high-risk patients, the following measures may be considered: (1) Delaying surgery and adjusting cardiac function; (2) Strengthening myocardial protection and reducing afterload intraoperatively, such as replacing cardioplegia, choosing palliative surgery to preserve more subvalvular structures, and reducing CPB and operation time; (3) Delaying extubation, strengthening sedation, reducing oxygen consumption, and enhancing cardiac output including vasoactive drugs or (and) mechanical assistance, postoperatively.

### The postoperative model timely considers increased LCOS risk during surgery

To explore the impact of surgical factors on LCOS risk, the postoperative model contained enriched variables during the intraoperative and early postoperative period in addition to preoperative variables. Among the output variables of the postoperative model, the ejection fraction replaced age as the primary factor. The duration of poor MVP and high CVP were also risk factors for LCOS in the model, reflecting insufficient arterial perfusion dynamics [30] and high right ventricular preload [31]. The actime is also a reported LCOS risk factor [32]. Although the preoperative variables urea and total protein were not output in the preoperative model, they became meaningful factors in the postoperative model. To our knowledge, the predictive significance of these parameters for LCOS has not been reported.

Notably, as an independent predictor of LCOS [13], the importance of lactate obtained 0.5 h postoperatively was highest in the postoperative model. Therefore, to incorporate lactate, we constructed the postoperative model rather than the intraoperative model at the expense of the short time from intraoperative to 0.5 h postoperatively. As a novel tool to assess the occurrence of LCOS, our postoperative model considered the risk factors of valve surgery at each stage as comprehensively as possible in the early postoperative period, so as to timely take precautions, such as enhanced circulatory support and reduced oxygen consumption.

### Easy-to-use bedside application interface

The clinical use of predictive models has always been a challenge. Most physicians do not understand and have time to learn programming languages, so they cannot operate traditional predictive models. Our model has been visualized for bedside use, and the predicted LCOS probability (%) can be displayed by clicking the ‘Predict’ button after entering the data in the input module. The model will alarm when high-risk populations are predicted. The actual test took only 65.03–163.86 s from entering the variables to displaying the results. Therefore, everyone can easily operate our models at the bedside and obtain prediction results immediately.

### Comparison with previous tools

Common tools for evaluating LCOS are inotropic score (IS), VIS, and LCOS score (LCOSS). IS and VIS quantify the degree of hemodynamic support by calculating the maximum doses of vasoactive drugs and inotropes [22,33], of which the latter is a modified version of the former. LCOSS considers other variables that contribute to cardiovascular insufficiency based on VIS, including heart rate, urine output, toe temperature, volume management, near-infrared spectroscopy (NIRS) measurements, and lactate [34]. Unfortunately, these scoring systems not only have defects such as high dependence on subjective judgments of physicians, insufficient inclusion of drugs and risk factors, and poor interpretability [35], but also delayed treatment due to late evaluation (mostly 24 h postoperatively among adult cardiothoracic surgery). Compared with VIS score, our model predicts LCOS risk earlier, with higher AUC, interpretability, and comprehensiveness. LCOSS was not compared because some variables such as toe temperature and NIRS data were not readily available.

Although predictive models objectively assess risk based on multivariate with higher accuracy and timeliness than scoring tools, only one logistic regression model have been reported for preoperative and intraoperative prediction of LCOS after valve surgery [36]. Logistic regression models are programmed with assumptions about the relationship between input and output variables, which results in the absence of some statistically insignificant but critical variables [17]. Our model was built through big data learning instead of specific programming, and integrated different algorithms to make the results more objective and accurate. The sample size for modeling was also larger. Moreover, postoperative prediction included not only more intraoperative variables but also variables 0.5 h postoperatively, which fully considered the increased LCOS risk by surgery while ensuring timeliness. Therefore, our model is more suitable than scoring tools and existing models for predicting LCOS after valve surgery.

Several limitations should be acknowledged. First, the sample data used for modeling were from a single center. Nevertheless, this does not defeat the purpose of the study, which is to apply ML to predict the risk of LCOS after valve surgery, rather than to develop the ultimate clinical general-purpose model. With the import of data from other institutions in the future, our model will be applicable to a wider range. On the other hand, the model lacks external and prospective validation. External validation and prospective validation may be more important in evaluating the performance of our predictive models. However, we split the data into training and testing datasets in the ratio of 80% and 20%, so our model has been validated internally. Cross-validation was also performed during training. Besides, 89 patients missing key variables were excluded, possibly contributing to some risk factors not being considered. However, those patients were few and had a negligible impact on the results. Finally, the current study only focused on the occurrence of LCOS after valve surgery as the primary outcome, lacking consideration of secondary outcomes such as investigating in-hospital mortality and length of stay in intensive care units and hospitals. They contribute to identify clinical outcomes in patients with LCOS [37] and further build death prediction models, which will be a direction for our future research.

## Conclusions

In this study, we constructed the first interpretative ML tool for on-line early predicting LCOS risk after valve surgery in critical care, in which the SVM model has the best performance. The tool contains two prediction periods, preoperatively and 0.5 h postoperatively, with great AUC, calibration capability, timeliness, interpretability, and clinical friendliness. Our tool could assist clinicians in early detection of high-risk patients of LCOS during postoperative critical care for intervention and prevention of the occurrence of LCOS, which requires external validation of multicenter cohorts in future clinical applications.

## Supplementary Material

Supplemental Material

## Data Availability

The original contributions presented in this study are included in the Supplementary materials, further inquiries can be directed to the corresponding authors.

## Central picture

Machine learning models show good performance for predicting low cardiac output syndrome.

## Central message

We developed machine learning models for predicting low cardiac output syndrome associated with valve surgery, which performed well and were interpreted and deployed online.

## Perspective statement

The low cardiac output syndrome complicating valve surgery leads to poor outcomes and lacks reliable means for early recognition. We developed machine learning models for predicting the complication preoperatively and 0.5h postoperatively, which performed well and have been interpreted and deployed online, reserving enough time for early intervention.

## References

[1] Whitson BA. Commentary: low cardiac output syndrome: a definition or a diagnosis code? J Thorac Cardiovasc Surg. 2022;163(5):1–12. doi: 10.1016/j.jtcvs.2020.09.040. 33019969

[2] Balderas-Munoz K, Rodriguez-Zanella H, Fritche-Salazar J, et al. Improving risk assessment for post-surgical low cardiac output syndrome in patients without severely reduced ejection fraction undergoing open aortic valve replacement. The role of global longitudinal strain and right ventricular free wall strain. Int J Cardiovasc Imaging. 2017;33:1483–1489. 28488096 10.1007/s10554-017-1139-6

[3] Pieri M, Belletti A, Monaco F, et al. Outcome of cardiac surgery in patients with low preoperative ejection fraction. BMC Anesthesiol. 2016;16(1):97. doi: 10.1186/s12871-016-0271-5. 27760527 PMC5069974

[4] Yotti R, Bermejo J, Gutiérrez-Ibañes E, et al. Systemic vascular load in calcific degenerative aortic valve stenosis: insight from percutaneous valve replacement. J Am Coll Cardiol. 2015;65(5):423–433. doi: 10.1016/j.jacc.2014.10.067. 25660919

[5] Mazhar R. Possible role of juxtaglomerular apparatus in low cardiac output syndrome and multiple organ failure: modulation by high sodium load. Med Hypotheses. 2001;57(1):128–130. doi: 10.1054/mehy.2000.1223. 11421643

[6] Hijazi RM, Sessler DI, Liang C, et al. Association between in-hospital mortality and low cardiac output syndrome with morning versus afternoon cardiac surgery: a retrospective cohort study. Anesthesiology. 2021;134(4):552–561. doi: 10.1097/ALN.0000000000003728. 33592096

[7] Uhlig K, Efremov L, Tongers J, et al. Inotropic agents and vasodilator strategies for the treatment of cardiogenic shock or low cardiac output syndrome. Cochrane Database Syst Rev. 2020;2020(11):CD009669. doi: 10.1002/14651858.CD009669.pub4. PMC8094388 33152122

[8] Landoni G, Lomivorotov VV, Alvaro G, et al. Levosimendan for hemodynamic support after cardiac surgery. N Engl J Med. 2017;376(21):2021–2031. doi: 10.1056/NEJMoa1616325. 28320259

[9] Chung J, Goldhammer JE. Con: preoperative echocardiography should be reviewed before cardiac surgery. J Cardiothorac Vasc Anesth. 2020;34(3):830–831. doi: 10.1053/j.jvca.2019.10.032. 31812563

[10] Juhl-Olsen P, Smith SH, Grejs AM, et al. Automated echocardiography for measuring and tracking cardiac output after cardiac surgery: a validation study. J Clin Monit Comput. 2020;34(5):913–922. doi: 10.1007/s10877-019-00413-w. 31677135

[11] Hadian M, Kim HK, Severyn DA, et al. Cross-comparison of cardiac output trending accuracy of LiDCO, PiCCO, FloTrac and pulmonary artery catheters. Crit Care. 2010;14(6):R212. doi: 10.1186/cc9335. 21092290 PMC3220011

[12] Ameloot K, Palmers P-J, Malbrain ML. The accuracy of noninvasive cardiac output and pressure measurements with finger cuff: a concise review. Curr Opin Crit Care. 2015;21(3):232–239. doi: 10.1097/MCC.0000000000000198. 25922896

[13] Rao V, Ivanov J, Weisel RD, et al. Lactate release during reperfusion predicts low cardiac output syndrome after coronary bypass surgery. Ann Thorac Surg. 2001;71(6):1925–1930. doi: 10.1016/s0003-4975(01)02634-0. 11426770

[14] Farag M, Veres G, Szabó G, et al. Hyperbilirubinaemia after cardiac surgery: the point of no return. ESC Heart Fail. 2019;6(4):694–700. doi: 10.1002/ehf2.12447. 31095903 PMC6676269

[15] Hong L, Xu H, Ge C, et al. Prediction of low cardiac output syndrome in patients following cardiac surgery using machine learning. Front Med. 2022;9:973147. doi: 10.3389/fmed.2022.973147. PMC9448978 36091676

[16] Meyer A, Zverinski D, Pfahringer B, et al. Machine learning for real-time prediction of complications in critical care: a retrospective study. Lancet Respir Med. 2018;6(12):905–914. doi: 10.1016/S2213-2600(18)30300-X. 30274956

[17] Tseng P-Y, Chen Y-T, Wang C-H, et al. Prediction of the development of acute kidney injury following cardiac surgery by machine learning. Crit Care. 2020;24(1):478. doi: 10.1186/s13054-020-03179-9. 32736589 PMC7395374

[18] Gregory A, Stapelfeldt WH, Khanna AK, et al. Intraoperative hypotension is associated with adverse clinical outcomes after noncardiac surgery. Anesth Analg. 2021;132(6):1654–1665. doi: 10.1213/ANE.0000000000005250. 33177322 PMC8115733

[19] Batista GE, Monard MC. An analysis of four missing data treatment methods for supervised learning. Appl Artif Intel. 2003;17(5–6):519–533. doi: 10.1080/713827181.

[20] Shalabi LA, Shaaban Z, Kasasbeh B. Data mining: a preprocessing engine. J Comput Sci. 2006;2(9):735–739. doi: 10.3844/jcssp.2006.735.739.

[21] Okada S, Ohzeki M, Taguchi S. Efficient partition of integer optimization problems with one-hot encoding. Sci Rep. 2019;9(1):13036. doi: 10.1038/s41598-019-49539-6. 31506502 PMC6737027

[22] Koponen T, Karttunen J, Musialowicz T, et al. Vasoactive-inotropic score and the prediction of morbidity and mortality after cardiac surgery. Br J Anaesth. 2019;122(4):428–436. doi: 10.1016/j.bja.2018.12.019. 30857599 PMC6435836

[23] Pérez Vela JL, Jiménez Rivera JJ, Alcalá Llorente MÁ, et al. Low cardiac output syndrome in the postoperative period of cardiac surgery. Profile, differences in clinical course and prognosis. The ESBAGA study. Med Intensiva. 2018;42(3):159–167. doi: 10.1016/j.medine.2018.03.001. 28736085

[24] Maganti MD, Rao V, Borger MA, et al. Predictors of low cardiac output syndrome after isolated aortic valve surgery. Circulation. 2005;112(9 Suppl):I448–I452. doi: 10.1161/CIRCULATIONAHA.104.526087. 16159861

[25] Maganti M, Badiwala M, Sheikh A, et al. Predictors of low cardiac output syndrome after isolated mitral valve surgery. J Thorac Cardiovasc Surg. 2010;140(4):790–796. doi: 10.1016/j.jtcvs.2009.11.022. 20152992

[26] Lomivorotov VV, Efremov SM, Kirov MY, et al. Low-cardiac-output syndrome after cardiac surgery. J Cardiothorac Vasc Anesth. 2017;31(1):291–308. doi: 10.1053/j.jvca.2016.05.029. 27671216

[27] Wu WY, Biery DW, Singh A, et al. Recovery of left ventricular systolic function and clinical outcomes in young adults with myocardial infarction. J Am Coll Cardiol. 2020;75(22):2804–2815. doi: 10.1016/j.jacc.2020.03.074. 32498808 PMC7392115

[28] Narayanan K, Reinier K, Teodorescu C, et al. Left ventricular diameter and risk stratification for sudden cardiac death. J Am Heart Assoc. 2014;3(5):e001193. doi: 10.1161/JAHA.114.001193. 25227407 PMC4323796

[29] Kalbacher D, Tigges E, Boekstegers P, et al. Underweight is associated with inferior short and long-term outcomes after MitraClip implantation: results from the German TRAnscatheter mitral valve interventions (TRAMI) registry. Am Heart J. 2020;222:73–82. doi: 10.1016/j.ahj.2019.12.022. 32018204

[30] Evans L, Rhodes A, Alhazzani W, et al. Surviving sepsis campaign: international guidelines for management of sepsis and septic shock 2021. Intensive Care Med. 2021;47(11):1181–1247. doi: 10.1007/s00134-021-06506-y. 34599691 PMC8486643

[31] Berlin DA, Bakker J. Starling curves and central venous pressure. Crit Care. 2015;19(1):55. doi: 10.1186/s13054-015-0776-1. 25880040 PMC4329649

[32] Umakanthan R, Leacche M, Petracek MR, et al. Safety of minimally invasive mitral valve surgery without aortic cross-clamp. Ann Thorac Surg. 2008;85(5):1544–1550. doi: 10.1016/j.athoracsur.2008.01.099. 18442535

[33] Gupta P, Rettiganti M, Wilcox A, et al. An empirically derived pediatric cardiac inotrope score associated with pediatric heart surgery. In: Seminars in thoracic and cardiovascular surgery. Elsevier; 2018;30(1):62–68. doi: 10.1053/j.semtcvs.2018.01.003. 29360599

[34] Ulate KP, Yanay O, Jeffries H, et al. An elevated low cardiac output syndrome score is associated with morbidity in infants after congenital heart surgery. Pediatr Crit Care Med. 2017;18(1):26–33. doi: 10.1097/PCC.0000000000000979. 28060152

[35] Alten JA, Gaies M. Defining low cardiac output syndrome: an ode to justice Potter Stewart. Pediatr Crit Care Med. 2017;18(1):85–87. doi: 10.1097/PCC.0000000000000989. 28060156

[36] Liu Y, Xiao J, Duan X, et al. The multivariable prognostic models for severe complications after heart valve surgery. BMC Cardiovasc Disord. 2021;21(1):491. doi: 10.1186/s12872-021-02268-z. 34635052 PMC8504034

[37] Zhao X, Gu B, Li Q, et al. Machine learning approach identified clusters for patients with low cardiac output syndrome and outcomes after cardiac surgery. Front Cardiovasc Med. 2022;9:962992. doi: 10.3389/fcvm.2022.962992. 36061544 PMC9434347

