# Supplementary material for: A novel interpretative tool for early prediction of low cardiac output syndrome after valve surgery: online machine learning models
Source: Ann Med. 2023 Dec 21;55(2):2293244. doi: 10.1080/07853890.2023.2293244 (PMC10763875; doi:10.1080/07853890.2023.2293244)
Supplement: Supplemental Material [file IANN_A_2293244_SM6686.docx]

# Additional File

**A novel interpretative tool for early prediction of low cardiac output syndrome after valve surgery: Online machine learning models**

Liang Hong^1#^, Tianling Feng^2,3#^, Runze Qiu^3,4#,^*, Shiteng Lin^2,3#^, Yinying Xue^1^, Kaizong Huang^3,4^, Chen Chen^3,4^, Jiawen Wang^3,5^, Rongrong Xie^1^, Sanbing Song^1^, Cui Zhang^1^* , Jianjun Zou^3,4^*

^1^Cardiovascular Intensive Care Unit, Department of Critical Care Medicine, Nanjing First Hospital, Nanjing Medical University, Nanjing 210006, China

^2^School of Basic Medicine and Clinical Pharmacy, China Pharmaceutical University, Nanjing 211198, China;

^3^Department of Clinical Pharmacology, Nanjing First Hospital, Nanjing Medical University, Nanjing 210006, China.

^4^Department of Pharmacy, Nanjing First Hospital, China Pharmaceutical University, Nanjing 210006, China.

^5^School of Pharmacy, Nanjing University of Chinese Medicine, Nanjing 210023, China.

*CORRESPONDENCE:

Cui Zhang, [zhangcuiacc@163.com](mailto:zhangcuiacc@163.com)；

Runze Qiu, rz_qiu@yeah.net；

Jianjun Zou, [zoujianjun100@126.com](mailto:zoujianjun100@126.com)

**
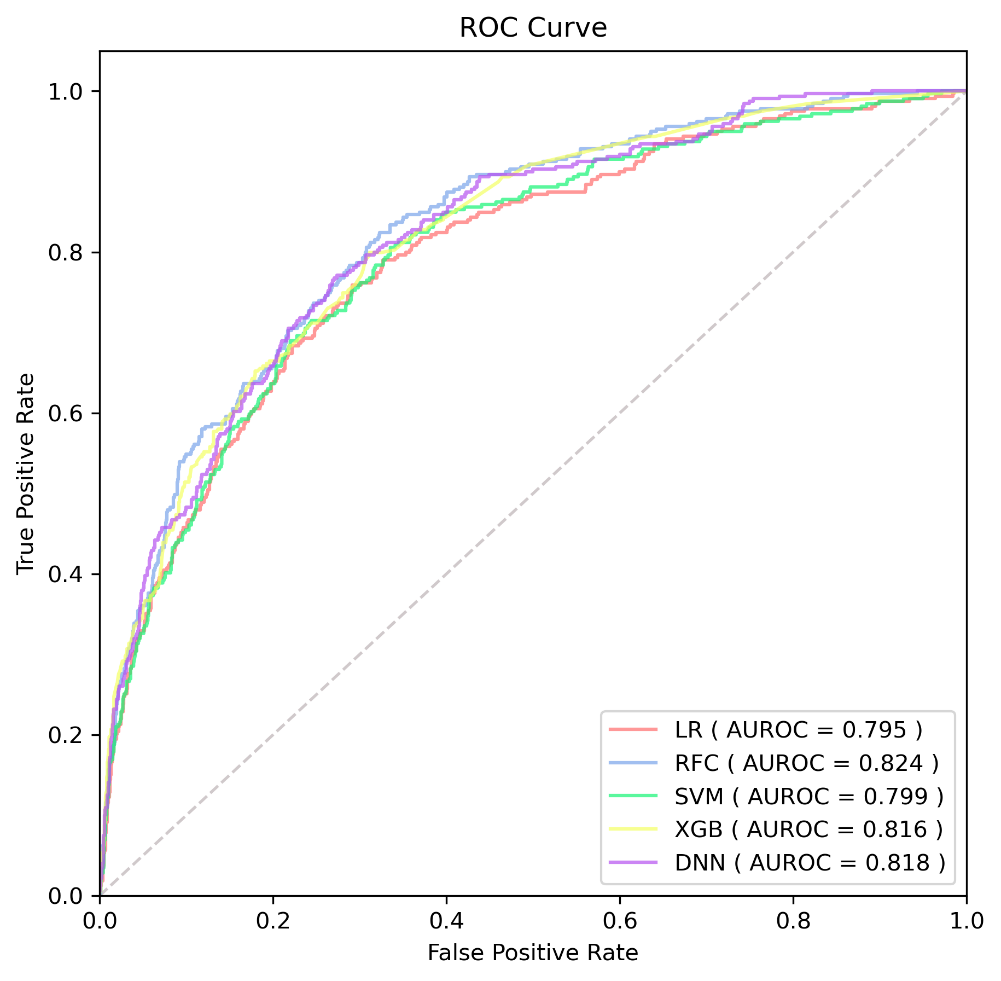
**

**e-Figure 1** The receiver operating characteristic curve (ROC) of the preoperative models on training cohort

**
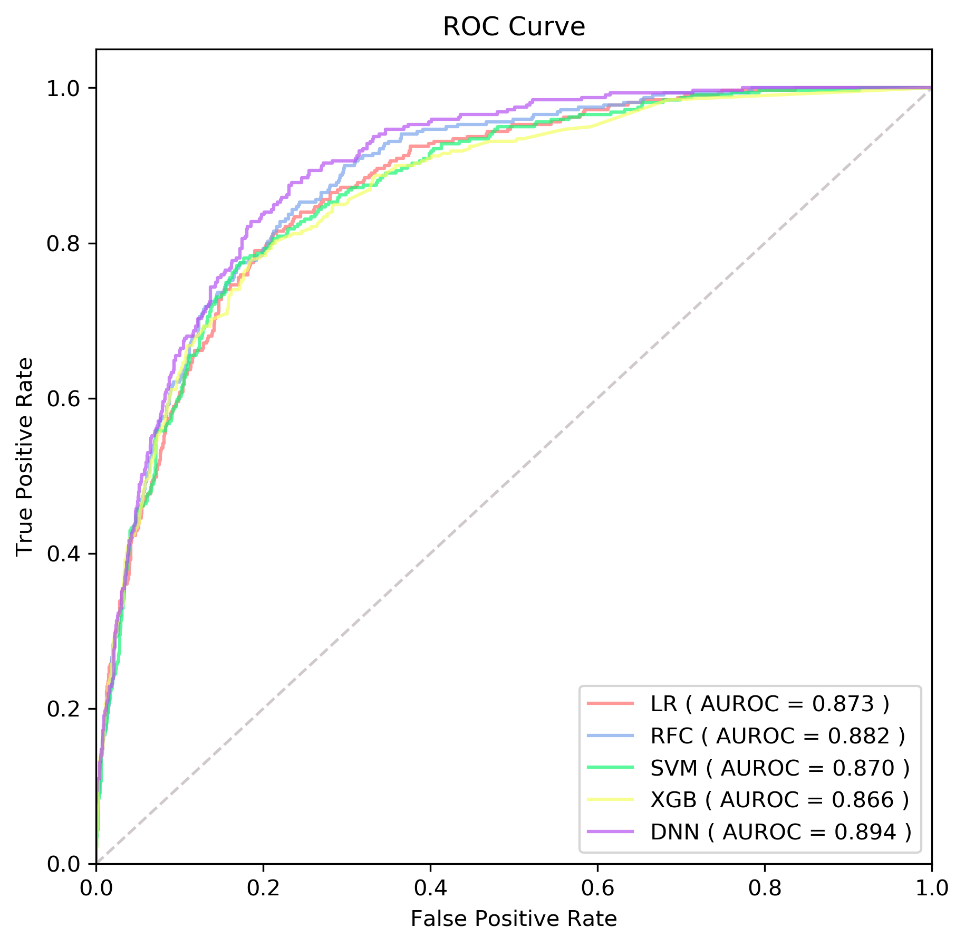
**

**e-Figure 2.** The receiver operating characteristic curve (ROC) of the postoperative models on training cohort
